# Associations between suicidal ideation, depression and substance use in university students

**DOI:** 10.1192/j.eurpsy.2026.10694

**Published:** 2026-07-31

**Authors:** E. B. Camargo Júnior, A. Busatta, M. N. D. F. Fernandes, L. B. H. Gimenez, R. C. D. D. Silva, G. G. Silva, E. C. D. S. Gherardi-Donato

**Affiliations:** 1 University of Rio Verde, Rio Verde; 2 Federal University of Maranhão, Imperatriz; 3 Federal University of Mato Grosso, Sinop; 4 Ribeirão Preto School of Nursing – EERP University of São Paulo – USP, Ribeirão Preto, Brazil

## Abstract

**Introduction:**

Suicidal ideation is a multifactorial and concerning phenomenon among university students, especially in contexts marked by academic challenges, emotional vulnerability, and substance use.

**Objectives:**

This study aimed to identify the prevalence of suicidal ideation and its association with depression and substance use among Brazilian university students.

**Table 1.** Multivariate logistic regression models for factors associated with suicidal ideation among university students. Table 1. Long description.

Variable	Model 1aOR (95% CI)	p-value	Model 2aOR (95% CI)	p-value
Depression (present)	24.6 (12.9 – 46.8)	< 0.001	43.34 (5.57 – 337.01)	< 0.001
Tobacco (moderate/high risk)	2.7 (1.43 – 5.24)	0.002	2.73 (1.05 – 7.06)	0.038
Alcohol (moderate/high risk)	2.3 (1.4 – 3.9)	< 0.001	1.06 (0.42 – 2.66)	0.895
Illicit drugs (moderate/high risk)	1.58 (0.89 – 2.80)	0.11	0.98 (0.41 – 2.38)	0.981

*Model 1 – adjusted for sex, sexual orientation, and religion. Model 2 – adjusted for sex, sexual orientation, religion, depression, and use of tobacco, alcohol, and illicit drugs.*

**Methods:**

This is a cross-sectional study with a convenience sample composed of students from a multicampus university located in five municipalities in the state of Goiás, Brazil. Participants were undergraduate students aged 18 years or older. Data collection was conducted at the beginning of each academic semester through, self-administered online questionnaire, applied at three time points (Feb/2021, Jul/2021, and Feb/2022). Suicidal ideation was assessed using item 9 of the *Patient Health Questionnaire-9 (PHQ-9).* Depression was measured using the total PHQ-9 score, with a cutoff point of ≥10. Substance use was assessed using the *Alcohol, Smoking and Substance Involvement Screening Test (ASSIST)*, with participants classified as low or moderate/high risk based on their scores. The study was approved by the Research Ethics Committee (protocol no. 4.434.811).

**Results:**

The sample consisted of 1,271 university students, predominantly women, heterosexuals, single individuals and white. The prevalence of suicidal ideation was 10.14%. The prevalence of depression was 33.4%, with a significant association with suicidal ideation (27.8%; p < 0.001). Bivariate logistic regression indicated a higher likelihood of suicidal ideation among women (OR = 2.61; p < 0.001), sexual minority students (OR = 3.49; p < 0.001), and those with moderate/high-risk alcohol consumption (OR = 2.43; p < 0.001). Depression showed the strongest association, with a 29.3-fold increase in the odds of suicidal ideation (p < 0.001). In the multivariate analysis (Model 2) depression (aOR = 43.34; p < 0.001) and moderate/high-risk tobacco use (aOR = 2.73; p = 0.038) remained significantly associated with suicidal ideation.

**Conclusions:**

Suicidal ideation showed high prevalence and was associated with depression and tobacco use. The findings underscore the importance of implementing institutional strategies for mental health screening and care.

**Disclosure of Interest:**

None Declared

## Long descriptions

### Table 1. Long description

The table consists of five columns: Variable, Model 1 a O R (95% C I), p-value, Model 2 a O R (95% C I), and p-value.

* Depression (present): Model 1 shows an a O R of 24.6 (12.9 to 46.8) with a p-value less than 0.001. Model 2 shows an a O R of 43.34 (5.57 to 337.01) with a p-value less than 0.001.

* Tobacco (moderate/high risk): Model 1 shows an a O R of 2.7 (1.43 to 5.24) with a p-value of 0.002. Model 2 shows an a O R of 2.73 (1.05 to 7.06) with a p-value of 0.038.

* Alcohol (moderate/high risk): Model 1 shows an a O R of 2.3 (1.4 to 3.9) with a p-value less than 0.001. Model 2 shows an a O R of 1.06 (0.42 to 2.66) with a p-value of 0.895.

* Illicit drugs (moderate/high risk): Model 1 shows an a O R of 1.58 (0.89 to 2.80) with a p-value of 0.11. Model 2 shows an a O R of 0.98 (0.41 to 2.38) with a p-value of 0.981.

Model 1 is adjusted for sex, sexual orientation, and religion. Model 2 is adjusted for sex, sexual orientation, religion, depression, and use of tobacco, alcohol, and illicit drugs.

Navigate back to Table 1..

